# In vitro activities of novel 4-HPR derivatives on a panel of rhabdoid and other tumor cell lines

**DOI:** 10.1186/1475-2867-11-34

**Published:** 2011-09-27

**Authors:** Melissa E Smith, Bhaskar C Das, Ganjam V Kalpana

**Affiliations:** 1 Department of Genetics, Albert Einstein College of Medicine, 1300 Morris Park Avenue, Bronx, NY, 10461, USA; 2 Department of Nuclear Medicine, Albert Einstein College of Medicine, 1300 Morris Park Avenue, Bronx, NY, 10461, USA; 3 Department of Developmental and Molecular Biology, Albert Einstein College of Medicine, 1300 Morris Park Avenue, Bronx, NY, 10461, USA; 4 Albert Einstein Cancer Center, Albert Einstein College of Medicine, 1300 Morris Park Avenue, Bronx, NY, 10461, USA; 5 Department of Microbiology and Immunology, Albert Einstein College of Medicine, 1300 Morris Park Avenue, Bronx, NY, 10461, USA

## Abstract

**Background:**

Rhabdoid tumors (RTs) are aggressive pediatric malignancies with poor prognosis. N-(4-hydroxy phenyl) retinamide (4-HPR or fenretinide) is a potential chemotherapeutic for RTs with activity correlated to its ability to down-modulate Cyclin D1. Previously, we synthesized novel halogen-substituted and peptidomimetic-derivatives of 4-HPR that retained activity in MON RT cells. Here we analyzed the effect of 4-HPR in inhibiting the growth of several RT, glioma, and breast cancer cell lines and tested their effect on cell cycle, apoptosis and Cyclin D1 expression.

**Methods:**

Effect of compounds on RT cell cycle profiles, and cell death were assessed by MTS cell survival assays and FACS analysis. The effects of treatment on Cyclin D1 expression were determined by immunoblotting. The efficacy of these compounds on glioma and breast cancer cell lines was also determined using MTS assays.

**Results:**

Low micromolar concentrations of 4-HPR derivatives inhibited cell survival of all RT cells tested. The 4-HPR derivatives altered RT cell cycle profiles and induced high levels of cell death that was correlated with their potency. ATRA exhibited high IC_50 _values in all cell lines tested and did not cause cell death. In MON RT cells, the iodo-substituted compounds were more active than 4-HPR in inducing cell cycle arrest and apoptosis. Additionally, the activity of the compounds correlated with their ability to down-modulate Cyclin D1: while active compounds reduced Cyclin D1 levels, inactive ATRA did not. In glioma and breast cancer cell lines, 4-HPR and 4-HPR derivatives showed variable efficacy.

**Conclusions:**

Here we demonstrate, for the first time, that the inhibitory activities of novel halogen-substituted and peptidomimetic derivatives of 4-HPR are correlated to their ability to induce cell death and down-modulate Cyclin D1. These 4-HPR derivatives showed varied potencies in breast cancer and glioma cell lines. These data indicate that further studies are warranted on these derivatives of 4-HPR due to their low IC_50_s in RT cells. These derivatives are of general interest, as conjugation of halogen radioisotopes such as ^18^F, ^124^I, or ^131^I to 4-HPR will allow us to combine chemotherapy and radiotherapy with a single drug, and to perform PET/SPECT imaging studies in the future.

## Background

Rhabdoid tumors (RTs) are aggressive pediatric malignancies that occur within the kidneys, soft tissues, and central nervous system [1-4]. No standard or effective treatment strategies are currently available for these tumors and prognosis remains poor with a two-year survival rate of 10-25% [1,3-6]. Therefore, there is a need to develop novel therapeutic strategies for RTs. INI1 (hSNF5, BAF47, SMARCB1) is a tumor suppressor biallelically deleted in RTs. We have previously demonstrated that Cyclin D1 is a key target repressed by INI1 and is required for genesis and survival of RTs *in vitro *and *in vivo*. Furthermore, we have found that 4-HPR effectively inhibits RT growth and that the ability of 4-HPR to inhibit RTs is correlated to down-modulation of Cyclin D1 [7,8].

4-HPR is a synthetic retinoid that is well tolerated by humans and inhibits the growth of various cancer cells by inducing apoptosis and, in some cases, cell cycle arrest at various stages [9-13]. Inhibition of tumor cell growth by 4-HPR *in vitro *occurs at clinically achievable concentrations (IC_50_s ranging from 1 μM to 15 μM) [14]. 4-HPR is largely studied as a chemo-preventive agent in animal models of carcinogen-induced epithelial tumors and in patients at risk for breast cancer [10,11,15,16]. Additionally, treatment of pediatric neuroblastoma patients with 4-HPR has resulted in prolonged stabilization of disease in pilot clinical studies [17-20].

4-HPR induces apoptosis in tumor cells *in vitro *by various mechanisms including activating retinoic acid receptors (RAR) β and γ, inducing ceramide-dependent cytotoxicity, generating free radical oxygen species, inducing nitric oxide synthase expression resulting in nitric oxide-dependent cytotoxicity, and increasing the mitochondrial permeability transition [11,15,17,18,20]. 4-HPR also induces cell cycle arrest likely correlated with its ability to down-modulate proliferation-related targets such as c-Myc, telomerase, p34/cdc2, and Cyclin D1 [21,22]. Interestingly, over expression of Cyclin D1 sensitizes breast cancer cells to 4-HPR [23]. Based on this information, and the fact that RTs are dependent on Cyclin D1, we previously tested 4-HPR and found that it inhibits the growth of RTs *in vitro *and *in vivo *in a xenograft mouse model with efficacy correlated with down-modulation of Cyclin D1 [7].

Several reports indicate that synthetic analogs of 4-HPR are more active anti-tumor agents or have potentially reduced toxicities compared to 4-HPR. For example, *N*-benzyl hydroxyl retinamide, a non-hydrolysable carbon-linked analog does not suppress plasma vitamin A levels as 4-HPR does, and is therefore less toxic [24]. Additionally, conjugations of 4-HPR have exhibited increase anti-tumor activity [25,26].

Because derivatives of 4-HPR may demonstrate more potent biological activity, improved bioavailability, and/or reduced toxicities compared to 4-HPR itself, we previously synthesized several novel 4-HPR derivatives which are conjugated to halogens and other moieties, and peptidomimetic derivatives that replace the alkene backbone with a rigid ring structure to help increase compound stability and bioavailability *in vivo *[27,28]. In particular, the peptidomimetic derivatives are more lipophilic, which increases bioavailability and possibly facilitates crossing the blood-brain-barrier [29]. Additionally, the derivatives that have been conjugated to halogens may allow us to create radio-conjugates (i.e. 4-HPR conjugated to ^18^F, ^124^I, or ^131^I) that facilitate combining radiotherapy and chemotherapy in a single agent.

In this study we examined the activity of 4-HPR derivatives that we synthesized in the laboratory and tested their activity on RT cell lines and investigated their abilities to induce apoptosis, cell cycle arrest, and down-modulate Cyclin D1. Additionally, we defined the activity profiles of these compounds in several glioma and breast cancer cell lines, for which 4-HPR has also been suggested as a potential chemotherapeutic agent. We found that the 4-HPR derivatives tested potently inhibited RT cell survival with efficacy correlated with down-modulation of Cyclin D1. The derivatives tested were also active in inhibiting the growth of several glioma and breast cancer cell lines. Our results indicate that because of their low IC_50_s and their abilities to significantly down-modulate Cyclin D1 and induce apoptosis, halogen-conjugated and peptidomimetic derivatives of 4-HPR are likely to be excellent diagnostic and therapeutic agents for RTs. Additionally, the ability of the derivatives to significantly decrease the survival of glioma and breast cancer cell lines indicates that they may be of general interest for treating cancers that respond to 4-HPR or depend on Cyclin D1 for growth.

## Results and Discussion

### Low micromolar concentrations of 4-HPR and its derivatives effectively inhibit rhabdoid tumor cell growth

**Figure 1:**
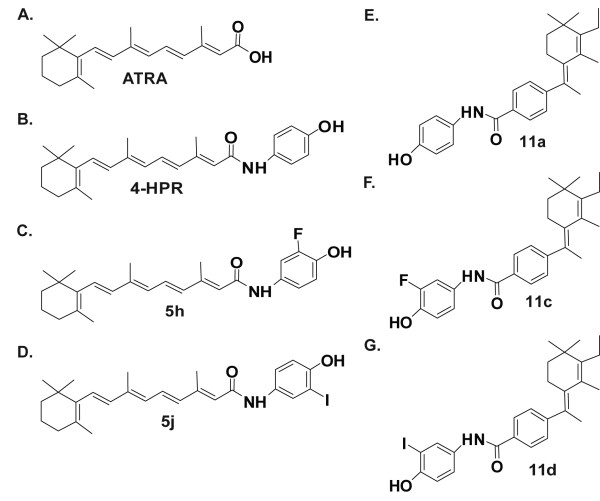
**Chemical structures of ATRA, 4-HPR, and 4-HPR derivatives and peptidomimetics**: A. ATRA, B. 4-HPR, C and D. 4-HPR derivatives, 5h (C) and 5j (D). E-G. 4-HPR peptidomimetics, 11a (E), 11c (F), and 11d (G).

**Figure 2:**
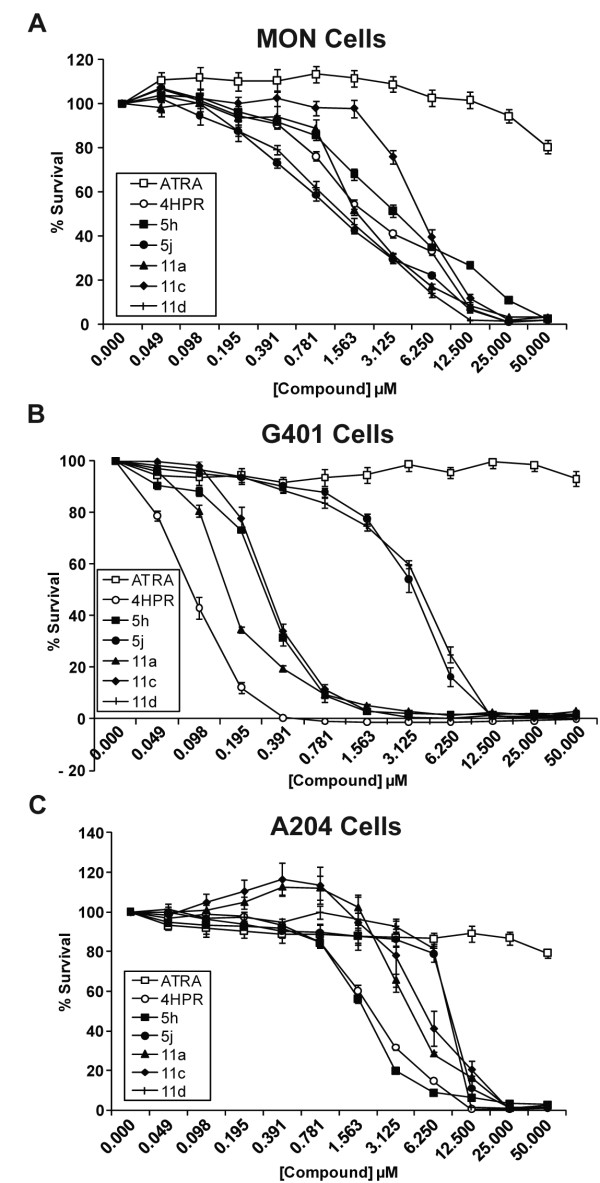
**Inhibition of RT cell growth by 4-HPR and its derivatives and peptidomimetics**: Survival curves of MON (A), G401 (B) and A204 (C) cells treated for three days with increasing concentrations of ATRA, 4-HPR, the most active halogen substituted derivative of 4-HPR (5h and 5j) and peptidomimetic compounds (11a, 11c and 11d).

Our previous studies indicated that halogen-substituted and peptidomimetic derivatives of 4-HPR have activity against one RT cell line, namely, MON [27,28]. These studies neither elucidated the mechanism of action of these new compounds nor tested activities on a wide range of RT or non-RT cell lines. Here we tested the efficacy of 4-HPR and highly active halogen-substituted (5h and 5j) and peptidomimetic derivatives (11a, 11c, and 11d) on three RT cell lines, MON, G401, and A204 (Figure 1). Since 4-HPR is derived from all trans retinoic acid (ATRA), which is used clinically as a differentiating and chemotherapeutic agent [30,31], we used ATRA as a control. The three cell lines cells were treated with increasing concentrations of indicated drugs and cell survival was determined. We found that ATRA did not show any activity in reducing the survival of RT cells, indicating that this compound is not a useful candidate for RT therapy (IC_50_s > 50 μM, Figure 2). 4-HPR potently inhibited the survival of RT cells with IC_50_s of 0.1 μM, 2.0 μM, and 2.2 μM in G401, A204, and MON cells respectively (Figure 2). Interestingly, iodo-derivatives of 4-HPR and the peptidomimetic compound tested, more potently decreased cell survival in MON cells with IC_50_s of 1.2 μM (5j) and 1.3 μM (11d) (Figure 2A). However, the derivatives and peptidomimetic compounds exhibited similar or less potent activities when compared to 4-HPR in G401 and A204 cells. A summary of IC_50_s of all the compounds in these three different RT cell lines is listed in Table 1.

**Table 1:** IC_50 _values for ATRA, 4-HPR, and derivatives on various tumor cell lines

	IC_50 _(μM)							
	
Tumor type Cell lines	Rhabdoid			Glioma			Breast	
	MON	G401	A204	SF268	U87	U251	MCF7	MDAMB231
ATRA	> 50	> 50	> 50	> 50	> 50	> 50	> 50	> 50
4-HPR	2.2	0.1	2.0	6.9	5.0	1.4	2.2	0.4
5h	3.6	0.3	1.7	18.9	16.3	5.8	6.3	N/A*
5j	1.2	3.0	8.2	12.0	13.7	9.1	11.8	23
11a	1.9	0.2	4.4	10.4	5.0	2.4	8.3	2.3
11c	5.2	0.3	5.6	15.7	9.3	5.9	12.1	5.3
11d	1.3	3.2	7.5	3.8	9.4	7.8	8.2	12.0

*The IC_50 _could not be determined because the survival curve was not sigmoidal

Our analysis indicated that the iodo-conjugated derivatives display significant potency in all RT cell lines tested. Since iodo conjugation can potentially be used to deliver radiotherapy and taken advantage of for radiation-based imaging (by substituting with ^124^I or ^131^I isotopes), these compounds are of great interest for further study. Hence we tested the ability of these compounds to induce cell cycle arrest and apoptosis, and to down-modulate Cyclin D1 in RT cells.

### Induction of cell cycle arrest in rhabdoid tumor cells lines treated with 4-HPR and its derivatives

**Figure 3:**
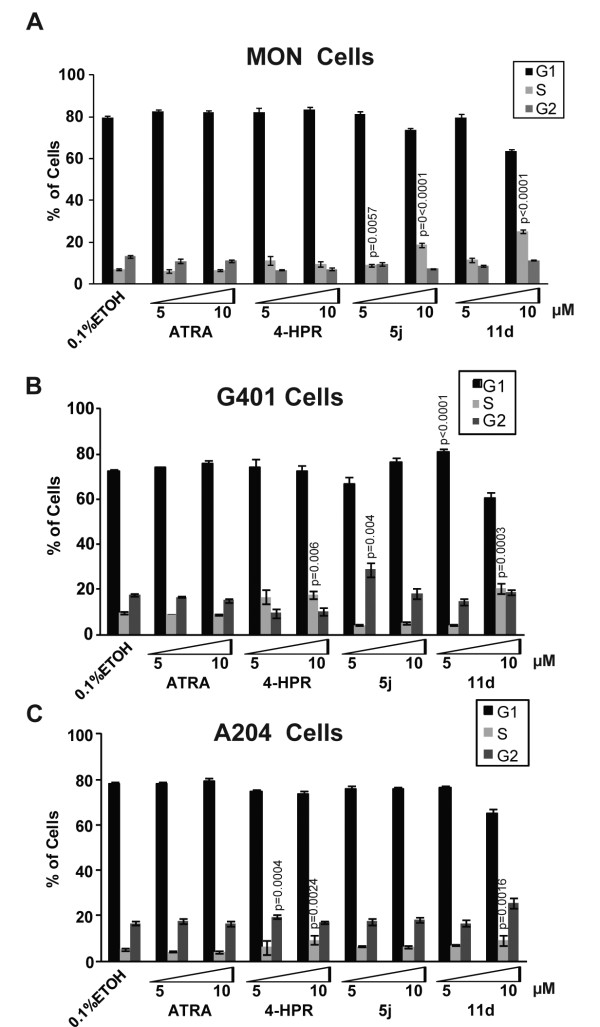
**Induction of cell cycle arrest by 4-HPR and its derivatives and peptidomimetics**: A-C. Cell cycle profile of MON (A), G401 (B), and A204 (C) cells treated for three days with 5 μM or 10 μM concentrations of ATRA, 4-HPR, and the iodo-conjugated 4-HPR derivative (5j) and peptidomimetic (11d).

To determine the effect of 4-HPR derivatives on the cell cycle profiles of RT cells we carried out FACS analysis of cells treated with 5 or 10 μM concentrations each of ATRA (as a control), 4-HPR, and its derivatives. ATRA did not induce cell cycle arrest in MON, G401, or A204 cells, consistent with the observation that this drug is not active against RT cells (Figure 3). We found that treatment with 4-HPR and its derivatives, however, induced complex changes in the cell cycle profiles of these cells. In MON cells, 4-HPR slightly increased the percentage of cells in S-phase, while both iodo-derivatives induced significant S-phase cell cycle arrest (P = 0.0057 for 5 μM 5j, P < 0.0001 for 10 μM 5j, P = 0.001 for 5 μM 11d, and P < 0.0001 for 10 μM 11d) (Figure 3A). Similarly, in G401 cells, 4-HPR increased the percentage of cells in S-phase at both 5 μM and 10 μM; however, S-phase arrest was only statistically significant at 10 μM (*P *= 0.006) (Figure 3B). The 4-HPR derivatives induced varying stages of cell cycle arrest in G401 cells: (i) 5 μM 5j induced G2-arrest (P = 0.004), (ii) 5 μM 11d induced G1-arrest (P < 0.0001), and (iii) 10 μM 11d induced S-arrest (P = 0.0003) (Figure 3B). In A204 cells 4-HPR caused G2-arrest at 5 μM (P = 0.0004) and S-arrest at 10 μM (P = 0.0024) (Figure 3C). Similarly to 4-HPR, 10 μM 11d induced S-arrest in A204 cells (P = 0.0016) (Figure 3C). In general, 4-HPR and its derivatives showed a tendency to significantly alter the cell cycle profiles of RT cells. Our results are consistent with previous observations of 4-HPR in other cancer cells, where this drug caused cell cycle arrest in G1, G2, and S phase [9,12,13].

### Induction of apoptosis in rhabdoid tumor cells by 4-HPR and 4-HPR derivatives and peptidomimetics

**Figure 4:**
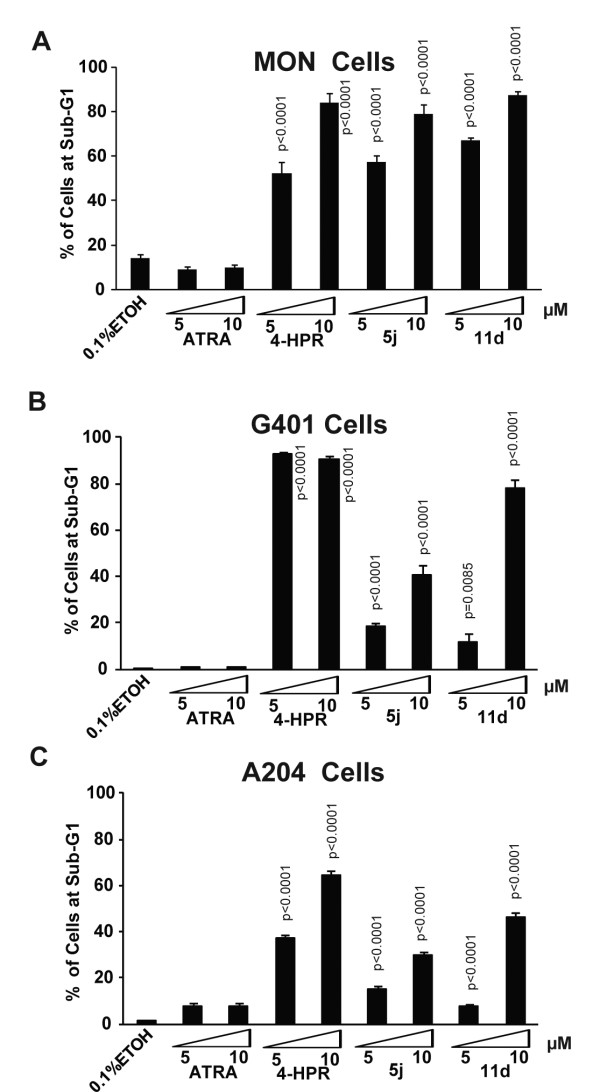
**Induction of cell death by 4-HPR and its derivatives and peptidomimetics**: A-C. Percentage of MON (A), G401 (B), and A204 (C) cells at sub- G_1 _when treated for three days with 5 μM or 10 μM concentrations of ATRA, 4-HPR, and the iodo-conjugated 4-HPR derivative (5j) and peptidomimetic (11d).

Though the nature of induction of cell-cycle arrest by 4-HPR and its derivatives varied from cell type to cell type, fluorescence activated cell sorting (FACS) analysis indicated that cell death, as measured by the percentage of cells in Sub G1/G0 stage, significantly and consistently increased in response to treatment in all cell lines tested. As expected, ATRA did not cause a significant increase in the percentage of sub-G1 cells above the background in MON and G401, but slightly increased the sub-G1 cells in A204 cells (*P *= 0.0076 at 5 μM and *P *= 0.0079 at 10 μM) (Figure 4A-C). On the other hand, 4-HPR and the iodo-conjugated derivatives (5j and 11d) significantly induced cell death in all 3 cells lines (*P *< 0.0001 for all drug treatment in all cells lines, except for 5 μM 11d in G401 cells with *P *= 0.0085) (Figure 4A-C). The level of cell death induced by the iodo-derivatives was similar to that induced by 4-HPR in MON cells (Figure 4A). In both G401 and A204 cells, however, the iodo-derivatives induced lower levels of cell death than did 4-HPR, consistent with the slightly increased IC_50 _values of the compounds in these 2 cell lines (Figure 4B and 4C). Taken together, our results indicated that 4-HPR and its iodo-conjugated derivatives induced cytotoxicity was correlated with their ability to stimulate high levels of cell death.

### Correlation of the activity of 4-HPR, 4-HPR derivatives and peptidomimetics to down-modulation of Cyclin D1 in RT cells

**Figure 5:**
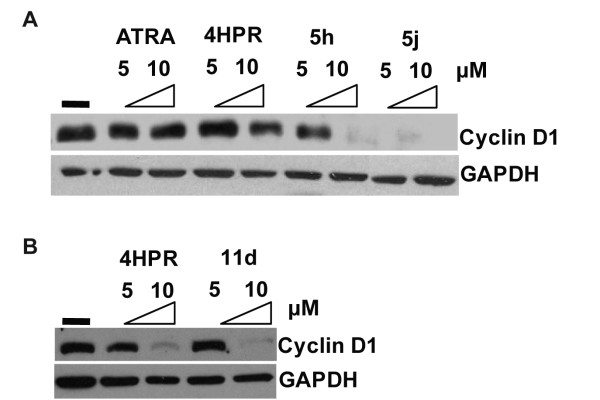
**Effects of 4-HPR and its derivatives and peptidomimetics on Cyclin D1 expression in MON cells**: A and B. Immunoblot analysis to determine the effect of 5 μM and 10 μM concentrations ATRA, 4-HPR, and 4-HPR derivatives and peptidomimetics on Cyclin D1 expression. GAPDH is used as a loading control.

Our previous studies indicated that RTs are exquisitely dependent on Cyclin D1 for their genesis and survival *in vitro *and *in vivo *[32]. Furthermore, the activity of 4-HPR in various cancer cell lines has been correlated with its ability to down-modulate Cyclin D1 [7,21,23,33]. Previous studies within our laboratory have also shown that the efficacy of 4-HPR in RT cells was correlated with down-modulation of Cyclin D1 [7]. For this reason, we sought to determine whether or not the inhibitory activities of 4-HPR derivatives were correlated with their ability to down-modulate Cyclin D1, and if the increased potency of some derivatives in MON cells could be explained by their improved ability to decrease Cyclin D1 levels. To test this, we carried out immunoblot analysis of whole cell lysates from MON cells before and after treatment with ATRA, 4-HPR, 5h, 5j, and 11d. We found that the efficacy of each compound was correlated with the ability to down-modulate Cyclin D1 protein levels. ATRA, as expected, did not decrease Cyclin D1 (Figure 5A and 5B). 4-HPR, which inhibits the survival of RT cells, caused a reduction in Cyclin D1 levels at 10 μM concentrations (Figure 5A and 5B). 4-HPR derivatives and peptidomimetics, which also exhibited IC_50_s in the low micromolar-range were able to down-modulate Cyclin D1 levels significantly, at both 5 and 10 μM concentrations, decreasing Cyclin D1 to undetectable levels at 10 μM (Figure 5A and 5B). These results indicated that the 4-HPR derivatives down-modulate Cyclin D1 at lower concentrations in MON cells showing that the chemical alterations made in the derivatives do not alter their ability to inhibit Cyclin D1.

### Inhibition of glioma and breast cancer cell growth by 4-HPR and 4-HPR derivatives and peptidomimetics

**Figure 6:**
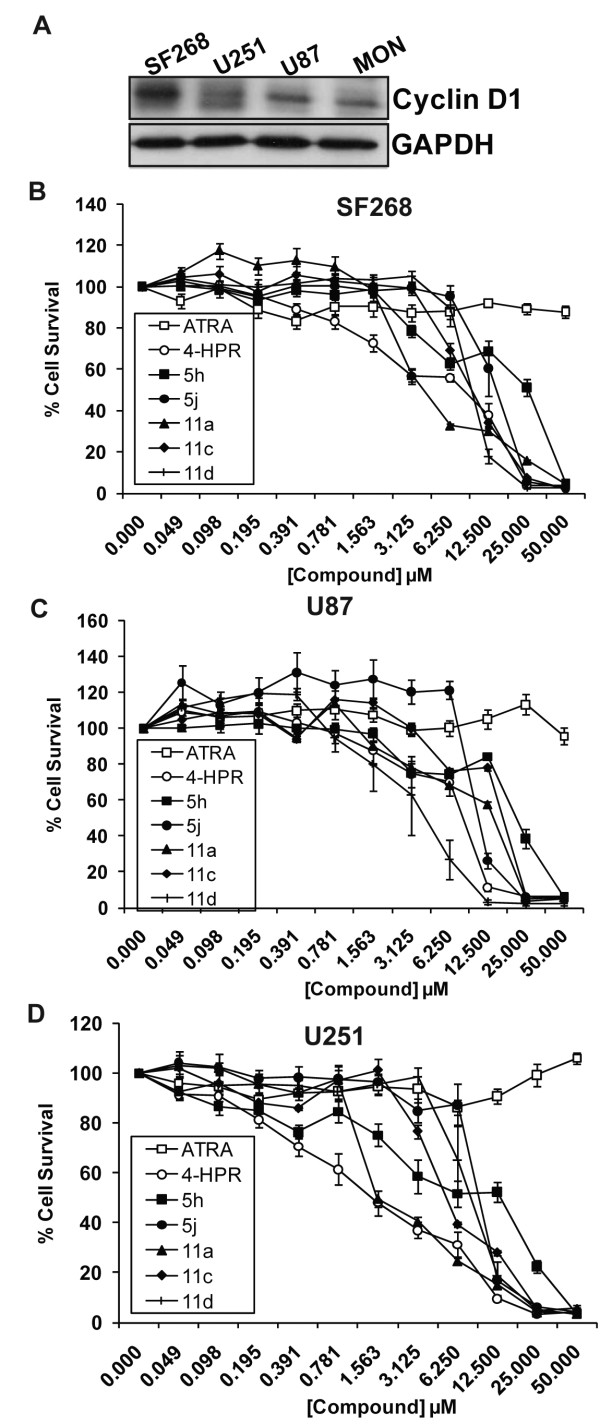
**Inhibition of glioma cell growth by 4-HPR and its derivatives and peptidomimetics**: A. Immunoblot analysis to determine the levels of Cyclin D1 protein expression in glioma cell lines compared to MON RT cells. B-D. Survival curves of SF268 (B), U87 (C), and U251 (D) glioma cells treated for three days with increasing concentrations of ATRA, 4-HPR, and 4-HPR derivatives and peptidomimetics.

**Figure 7:**
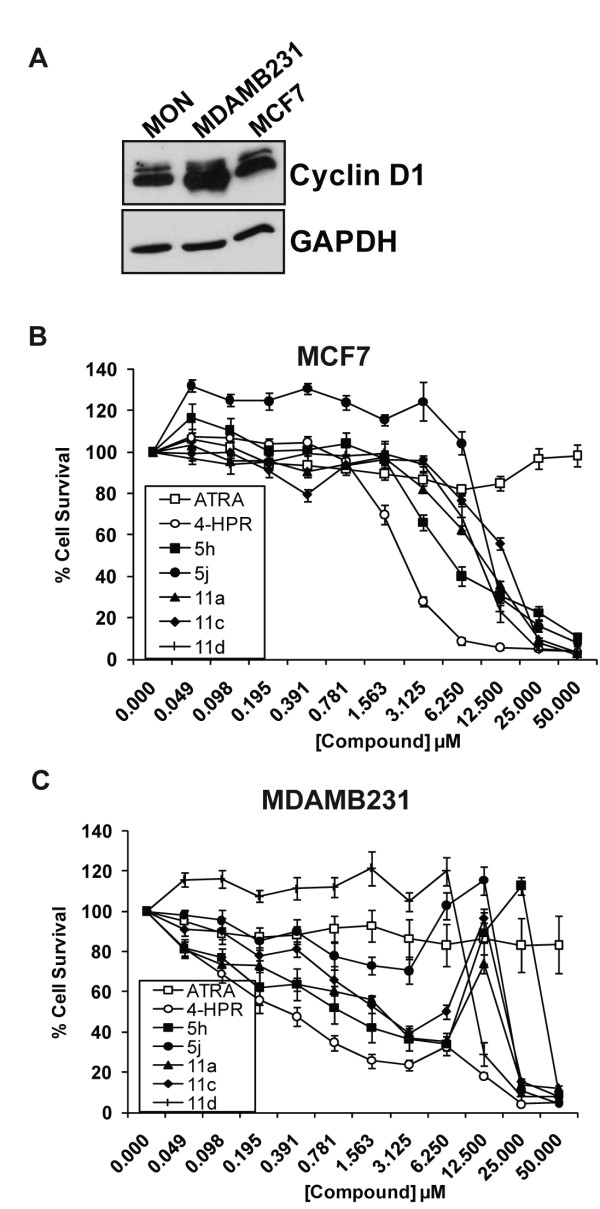
**Inhibition of breast cancer cell growth by 4-HPR and its derivatives and peptidomimetics**. A. Immunoblot analysis to determine the levels of Cyclin D1 protein expression in the breast cancer cell lines tested compared to MON RT cells. B and C. Survival curves of MCF7 (B) and MDAMB231 (C) cells treated for three days with increasing concentrations of ATRA, 4-HPR, and 4-HPR derivatives and peptidomimetics.

The activity of 4-HPR in glioma and breast cancer cell lines has been studied previously and 4-HPR has been indicated as a potential therapeutic for these 2 types of cancers [23,34-38]. In particular, 4-HPR has been implicated for use as a chemo-preventive agent for people at risk for breast cancer [10,16,39,40]. Additionally, many breast cancers and gliomas over-express Cyclin D1 [41,42]. We performed immunoblot analysis to determine the relative expression levels of Cyclin D1 in the glioma (SF268, U251, and U87) and breast cancer (MDAMB231 and MCF7) cell lines, when compared to RT cell lines such as MON. All cell lines tested expressed high levels of Cyclin D1; in some cases the level of Cyclin D1 was even higher than that found in MON RT cells (Figure 6A and 7A).

We tested the efficacy of 4-HPR derivatives in these glioma and breast cancer cell lines by treating cells with various concentrations of drugs for 3 days. We found that ATRA did not reduce the survival of these cells at concentrations of 50 μM and lower (Figures 6B-D and 7B and 7C). 4-HPR inhibited the growth of all glioma and breast cancer cell lines tested with IC_50_s ranging from 0.4 to 6.9 μM, suggesting that RT cells are more sensitive to 4-HPR than glioma and breast cancer cells. In general, the derivatives of 4-HPR were less effective than parent 4-HPR in inhibiting the growth of these non-RT cells. IC_50_s of the compounds in glioma and breast cancer cells ranged from 0.4 μM to 23.0 μM and are presented in Table 1.

## Conclusions

Our report demonstrates that low micromolar concentrations of halogen-substituted 4-HPR derivatives and peptidomimetics inhibit the growth of several RT cell lines with similar potency to 4-HPR. This data thus, suggests that these derivatives of 4-HPR retain the activity and, in some cases, improve potency. Treatment with 4-HPR, 5j, and 11d induced varied effects on the cell cycle in the three RT cell lines. This variability was not surprising: many studies have shown that 4-HPR alters the cell cycle profiles of tumor cells in complex ways, inducing G_1_, G_2_, and S phase cell cycle arrest under different conditions [9,12,13]. In addition to cell cycle arrest, 4-HPR and both of the iodo-derivatives induced high levels of cell death in all RT cell lines tested. The most dramatic induction of cell death occurred in G401 cells, in which both 5 μM and 10 μM concentrations of all compounds induced approximately 90% cell death. These results indicate that the efficacies of these drugs are most closely correlated with their abilities to induce cell death than their ability to induce cell cycle arrest. Additionally, the efficacy of the compounds in MON RT cells was correlated with potent down-modulation of Cyclin D1.

It is important to note that parent compound ATRA did not show significant activity in any of the tumor cells tested, rhabdoid or otherwise. Correspondingly, ATRA treatment did not result in cell cycle arrest, enhanced cell death, or down-modulation of Cyclin D1 in RT cells. ATRA is an FDA-approved compound and is clinically used to treat some cancers including promyelocytic leukemia [30,31]. Unfortunately, our results suggest that ATRA is not a possible therapeutic for treatment of RTs.

Since 4-HPR has been indicated as a potential therapeutic for both gliomas and breast cancers [23,34-38], we also tested the efficacy of these derivatives in several glioma and breast cancer cell lines. Parent compound ATRA, did not reduce survival of these cell types, while 4-HPR was able to reduce their survival with IC_50_'s ranging from 0.4 μM to 6.9 μM. Interestingly, in both glioma and breast tumor cell lines tested, 4-HPR was more potent than derivatives 5j and 11d, with the exception of compound 11d in SF268 glioma cells, which was more (1.8-fold) potent in inducing cell death compared to 4-HPR. However, it is interesting to note that, in general, glioma and breast cancer cells were less sensitive to 4-HPR compared to RT cells.

There are several reports indicating that derivatives of 4-HPR show similar or increased anti-tumor activity and, in some cases, show decreased toxicities [24,25,43]. A non-hydrolysable carbon-linked analogs of 4-HPR, *N*-benzyl hydroxyl retinamide, does not suppress plasma vitamin A levels as 4-HPR does and is therefore less toxic [24]. Glycosyl conjugates of 4-HPR have exhibited increased anti-tumor activity [25]. One of the metabolite of 4-HPR, 4-oxo-fenretinide, exhibited increased potency in causing G2-M arrest [26]. However, the compounds that we describe here are halogen conjugated and peptidomimetic compounds derived from 4-HPR. Our previous report, for the first time, described the synthesis and identification of these halogen-substituted and peptidomimetic derivatives of 4-HPR [27,28]. We sought to create derivatives of 4-HPR that are at least as active as 4-HPR itself in killing RT cells, but that are also amenable for further modification such as conjugation of radioactive moieties or nanoparticles. We believe that conjugation of radioactive moieties or nanoparticles are likely to increase the therapeutic potential of 4-HPR but might also lead to development of compounds for use in imaging diagnostic studies. To facilitate such development, we designed and tested several halogen-conjugated derivatives of 4-HPR [27].

Previous reports from our laboratory indicated that 4-HPR derivatives are indeed active against MON RT cells. The data presented in this report indicate that the halogen-substituted derivatives and peptidomimetic derivatives of 4-HPR are active against a variety of cancer cells. Furthermore, we find that the activities of these compounds are correlated to their ability to induce potent cell death and down-modulation of Cyclin D1 expression. These data are consistent with the idea that 4-HPR can be further modified to create therapeutic compounds that are more stable and are likely to have better bioavailability profiles. In addition, the fact that halogen-derivatives of 4-HPR retain their activity suggests that conjugating 4-HPR to radioactive halogens will be a valuable clinical tool. Modifying the current derivatives to contain radioactive halogens, such as ^18^F, ^124^I, or ^131^I may allow us to use these compounds as imaging agents using positron emission tomography (PET) and as agents to deliver combined chemotherapy and radiotherapy. Radioactive derivatives can also be used to evaluate bioavailability of 4-HPR in vivo, using non-invasive imaging. In addition, the peptidomimetic derivatives of 4-HPR (11a, 11c, and 11d) may be more stable and may have the potential to traverse the blood-brain barrier because of the substitution of the alkene backbone with a lipophilic ring structure. Because of these reasons, and because of the relatively high tolerance and low toxicity of 4-HPR, we propose that further studies on these 4-HPR derivatives are warranted.

## Methods

### Cell culture and drugs

The RT cell lines used, MON [44], G401 (American Type Culture Collection), and A204 (American Type Culture Collection) were maintained in RPMI supplemented with 10% fetal bovine serum, 50 U/mL penicillin, 50 μg/mL streptomycin, and 2 mM L-glutamine. Cell plating and drug treatment was performed in RPMI supplemented with 10% charcoal/dextran-adsorbed fetal bovine serum (HyClone), 50 U/mL penicillin, 50 μg/mL streptomycin, and 2 mM L-glutamine.

Glioma cell lines SF268, U87, and U251 and breast cancer cell lines MCF7 and MDAMB231 were maintained in DMEM supplemented with 10% fetal bovine serum, 50 U/mL penicillin, 50 μg/mL streptomycin, and 2 mM L-glutamine. Cell plating and drug treatment was performed in DMEM supplemented with 10% charcoal/dextran-adsorbed fetal bovine serum (HyClone), 50 U/mL penicillin, 50 μg/mL streptomycin, and 2 mM L-glutamine.

Stock solutions of all drugs were prepared at 10 mM concentration in 100% ethanol. Working solutions were prepared by diluting the stocks into cell culture medium so that the final concentration of ethanol remained constant in all wells of each assay. 4-HPR was purchased from ONBIO (CAS# 65646-68-6) and all derivatives were synthesized by the author Dr. Bhaskar C. Das as previously described [27,28].

### Cell survival methyl tetrazolium salt (MTS) assays

Aliquots of 8 × 10^3 ^cells/well were plated in 96-well plates for all cell types except for G401, which were plated at 6.5 × 10^3 ^cells/well and treated with serial dilutions of drugs for three days. Cell survival was determined using an MTS assay kit (CellTiter 96 Aqueous One Solution Cell Proliferation Assay Kit; Promega).

### Cell cycle profile and cell death analysis

Propidium iodide staining and FACS analysis was performed as previously described [45]. CellQuest Pro software (BD Biosciences) was used to elaborate data. Analysis of cell death was performed by gating for the sub-G_1 _population during FACS as previously described [7].

### Statistical analysis

Statistical analysis of the data was performed using GraphPad Prism (San Diego, CA). T-tests and/or one-way ANOVA were used to analyze differences in cell cycle profiles and induction of cell death. Relative IC_50 _values for survival data were calculated using the nonlinear regression curve fit with sigmoidal dose response (variable slope) function. Relative IC_50 _is the concentration giving a response exactly halfway between the fitted top and bottom of the survival curve when graphed as percent inhibition versus the log of compound concentration.

### Immunoblot analysis

Immunoblot analysis was carried out using α-Cyclin D1 (Thermo Scientific, Catalog#MS-210) and α-GAPDH (Chemicon International, Catalog#MAB374). Chemiluminescence detection was achieved using SuperSignal West Pico and Femto Chemiluminescence Substrate for GAPDH and Cyclin D1 respectively [Pierce, Catalog#34080 (Pico) or #34095 (Femto)].

## Abbreviations

4-HPR: N-(4-hydroxyphenyl)retinamide; ANOVA: analysis of variance; ATRA: All-trans retinoic acid; DMEM: Dulbecco's modified Eagle's medium; FACS: Fluorescence activated cell sorting; FDA: Food and Drug Administration; IC50: half maximal inhibitory concentration; MTS: methyl-tetrazolium salt; PET: positron emission tomography; RAR: Retinoic acid receptor; RPMI: Roswell Park Memorial Institute culture medium; RT(s): Rhabdoid Tumor(s); SDS-PAGE: sodium dodecyl sulfate polyacrylamide gel electrophoresis.

## Competing interests

The authors declare that they have no competing interests.

## Authors' contributions

MES participated in the design of the study, performed all experiments, analyzed and interpreted the data, and drafted the manuscript. BCD synthesized the 4-HPR derivatives used for this study. GVK conceived of the study, designed the experiments, analyzed and interpreted data, and corrected the manuscript. All authors read and approved the final manuscript.
